# Transplanting Marginal Organs in the Era of Modern Machine Perfusion and Advanced Organ Monitoring

**DOI:** 10.3389/fimmu.2020.00631

**Published:** 2020-05-12

**Authors:** Thomas Resch, Benno Cardini, Rupert Oberhuber, Annemarie Weissenbacher, Julia Dumfarth, Christoph Krapf, Claudia Boesmueller, Dietmar Oefner, Michael Grimm, Sefan Schneeberger

**Affiliations:** ^1^Department of Visceral, Transplant and Thoracic Surgery, Medical University of Innsbruck, Innsbruck, Austria; ^2^Department of Cardiac Surgery, Medical University of Innsbruck, Innsbruck, Austria

**Keywords:** transplantation, machine perfusion, marginal, graft, immunogenecity, immunomodulation, reconditioning, expanded criteria donor

## Abstract

Organ transplantation is undergoing profound changes. Contraindications for donation have been revised in order to better meet the organ demand. The use of lower-quality organs and organs with greater preoperative damage, including those from donation after cardiac death (DCD), has become an established routine but increases the risk of graft malfunction. This risk is further aggravated by ischemia and reperfusion injury (IRI) in the process of transplantation. These circumstances demand a preservation technology that ameliorates IRI and allows for assessment of viability and function prior to transplantation. Oxygenated hypothermic and normothermic machine perfusion (MP) have emerged as valid novel modalities for advanced organ preservation and conditioning. *Ex vivo* prolonged lung preservation has resulted in successful transplantation of high-risk donor lungs. Normothermic MP of hearts and livers has displayed safe (heart) and superior (liver) preservation in randomized controlled trials (RCT). Normothermic kidney preservation for 24 h was recently established. Early clinical outcomes beyond the market entry trials indicate bioenergetics reconditioning, improved preservation of structures subject to IRI, and significant prolongation of the preservation time. The monitoring of perfusion parameters, the biochemical investigation of preservation fluids, and the assessment of tissue viability and bioenergetics function now offer a comprehensive assessment of organ quality and function *ex situ*. Gene and protein expression profiling, investigation of passenger leukocytes, and advanced imaging may further enhance the understanding of the condition of an organ during MP. In addition, MP offers a platform for organ reconditioning and regeneration and hence catalyzes the clinical realization of tissue engineering. Organ modification may include immunological modification and the generation of chimeric organs. While these ideas are not conceptually new, MP now offers a platform for clinical realization. Defatting of steatotic livers, modulation of inflammation during preservation in lungs, vasodilatation of livers, and hepatitis C elimination have been successfully demonstrated in experimental and clinical trials. Targeted treatment of lesions and surgical treatment or graft modification have been attempted. In this review, we address the current state of MP and advanced organ monitoring and speculate about logical future steps and how this evolution of a novel technology can result in a medial revolution.

## Introduction

The velocity and significance of progress in transplantation started to decrease around the turn of the century. Up until that point, the number of transplantations performed was ever-increasing, and the outcomes continued to improve. The clinical benefit to the patient from the immediate and effective treatment of organ failure was defined by improved patient survival. Patient survival as an endpoint followed by graft survival as a surrogate and indicator of judicious organ attribution were the framework of significant momentum that succeeded in making transplantation a standard of care. This success was fueled, however, not only by the healthcare quality gain but also by a flourishing industry producing high-quality and expensive immunosuppressive medication—to be taken for life after transplantation (1–3).

The honeymoon period came to an end when both donor rates and short-term results stagnated. The established endpoints for clinical trials are difficult to improve in the short run after transplantation, and the field was slow in anticipating and responding to the changing circumstances—specifically the shortage of standard criteria donors (SCD). The field of transplantation now finds itself in very difficult circumstances: 90%+ patient and graft survival rates leave little room for improvement but also little room for error. At the same time, the conditions and circumstances in organ donation are radically changing, with increasing donor age and comorbidities and donation from non-heart-beating donors. In countries spearheading the evolution, donation after cardiac death (DCD) and extended criteria donor (ECD) organs make up more than 50% of the organ pool. With the pressure to maintain and further improve the excellent short- and long-term results, but working with very different resources, the most prominent immediate challenge is to better define and preserve, if not improve, organ quality in transplantation.

With this as a driving force, modalities to prolong and improve preservation together with the tools to assess organ quality and function are emerging as the new frontier. Beyond serving an immediate medical need, the technologies evolving herald a more fundamental potential for change in healthcare: with the extracorporeal long-term preservation and assessment of organs, the capability for human organ treatment and repair arises. Hence, 20 years after transplantation advanced to become a standard of care, the field has the potential to play a key role and add a chapter to healthcare once again (4–10).

In this review, we aim to address the current state of the field. A clarification of the terminology and the definitions of marginal organs, including an overview and comparison between the definitions for each organ, shall help to provide a better picture of the actual status. Considering the changing circumstances, the actual impacts of a condition on organ quality and function need to be addressed not only from the viewpoint of an empirical correlation. The individual parameters need to be further addressed for their reversibility and treatability. Building on this concept, the various modalities of MP in their different stages of development and actual benefit require a critical reflection. While the assessment of organ quality and function during extracorporeal perfusion is in its infancy, the growing body of data may help to eventually develop a comprehensive picture of the quality of the components of organs as relevant to transplantation and beyond. An important focus in this context is the assessment, definition, and relevance of the immune system of organs if isolated from the human body. Immunomodulation and immunomasking seem more realistic if attempted under the conditions of an isolated and perfused organ. The vision of a realization and refinement of the repair, treatment, and modification of organs is starting to trigger a boost in interest both in academia and in industry.

## Defining “Marginal” Organs

In the light of organ shortage, critical reflection on the expansion of the donor pool is essential. The definition of organs that are marginal for transplantation is often considered a well-defined entity. In reality, however, the definition is very different for each organ, and there is incongruence between the various definitions. It is clear and well-justifiable that different organs are considered to be extended criteria organs going by different criteria, since the relevance of one single factor may be very diverse. The assessment of the outcome after transplantation of extended criteria organs, however, needs to be seen and interpreted with reference to the diverse definitions used throughout the years.

### ECD Kidneys

The determination of ECD evolved from the term “marginal donors,” and much of the literature focuses on criteria for living donors. Since this is not the focus of this article, we will restrict the research to deceased donors and deceased donor organs.

It is well-known that a number of factors may impact the eventual outcome after transplantation. The definition of cadaveric kidney organs marginal for transplantation emerged from prioritization of and preference for factors that may outrank other contributors in magnitude and simplicity of assessment (11). In the deceased donor situation, the prior medical history is not completely known, and the exact determination of hypertension, for example, often remains incomplete regarding details such as manifestation, duration, or response to treatment. Some of the understanding of donor risk factors also stems from living donors (12), where early definitions of marginal donors relate to the risks for the recipient rather than the risk for the donor, which was only assessed and understood much later. Hence, the definition of marginal or ECD kidneys needs to be used with caution since the definitions used are building on a set of parameters that have not been formally established, validated, and re-validated.

Still, compared to other organ systems in SOT, a comparatively good definition of marginal or ECD has been established. Noteworthily, the term “marginal” should be avoided in favor of ECD, as “marginal” may be considered pejorative by the patients who receive them and also by the programs that transplant them (13).

The ECD criteria most widely used in kidney transplantation are the OPTN-approved criteria, as described by Metzger et al. (13). They are shown in Table 1.

**Table 1:** Commonly used “ECD” criteria for the kidney.

	**Donor age category (years)**				
**Donor condition**	**<10**	**10–39**	**40–49**	**50–59**	**≥60**
CVA+ HTN+ creatinine >1.5 md/dL				x	x
CVA+ HTN				x	x
CVA+ creatinine >1.5 md/dL				x	x
CVA+ creatinine >1.5 md/dL				x	x
CVA+ creatinine >1.5 md/dL					x
CVA					x
HTN					x
Creatinine >1.5 mg/dL					x

*CVA, cerebrovascular accident was the cause of death; HTN, history of hypertension*.

A helpful tool that combines such parameters is the Kidney Donor Risk Index (KDRI), which was generated by weighting the following factors into a single number in order to predict the risk of post-transplant graft failure: age, weight, height, race, history of hypertension, history of diabetes, cause of death, hepatitis C status, serum creatinine, and DCD. The association of KDRI with the outcome in the Eurotransplant senior program (ESP) was evaluated by Schamberger et al. (14). Kidney transplantation from Maastricht category-III donors after circulatory death (DCD), which introduces a higher risk of primary non-function and delayed graft function (DGF), has been added to ECD criteria (15). Furthermore, kidneys from a donor with an acute kidney injury before organ procurement correspond to marginal quality (16). Importantly, kidneys from donors with a history of extracorporal membrane oxygenation (ECMO) have not been addressed specifically but can be classified as marginal organs since deteriorated microperfusion can be assumed (17–19).

### ECD Livers

In liver transplantation, the term “marginal” does not refer to a number of distinct internationally accepted criteria that could characterize a graft, and nor does “ECD.” Instead, both terms encompass a variety of factors that have been observed to limit graft quality, but the impact of each of these factors remains to be defined. Therefore, several models for the assessment of the sum of such risk factors have been introduced (20). In this regard, valuable predictive quality of patient and graft survival at 3 months was achieved using the Survival Outcomes Following Liver Transplantation (SOFT) score (21). Death censored graft survival at 60 months follow-up was well-predicted by the Donor Risk Index (DRI; c-index 0.59) and Eurotransplant-DRI (c-index 0.58) (20).

Table 2 lists those criteria that overlap in the mentioned scoring systems and that are widely applied when defining “marginal” or “ECD” liver grafts (22, 23). Apart from clinical and metric parameters, these include histological criteria, since liver biopsies are considered helpful in order to evaluate the organ quality. Whereas, liver fibrosis has been reported to predict a high incidence of early graft failure (24), Liu et al. (25) suggested a macrovesicular steatosis of 30 percent as a cutoff value for marginal grafts.

**Table 2:** Common criteria defining “marginal” or “expanded criteria donor (ECD)” livers, as well as “ECD DCD” livers.

**DBD**	
Cardiac arrest (min)	>15
Prolonged hypotensive periods	<60 mmHg for >1 hr
Age (yrs)	>55
BMI (kg/m^2^)	>30
HBV	Positive
HBC	Positive
BMI (kg/m^2^)	79 (100.0)
Macrosteatosis (%)	>30
Hypernatriemia (mEq/L)	>155
ICU stay (days)	≥ 5 (mechanical ventilation)
Nosocomial infection	Positive blood cultures or pneumonia
Split liver	yes
AST (U/L)	>170
ALT (U/L)	>140
CIT (hrs)	>12
Vasopressor drug requirement	Dopamine dose >10 μg/kg/min or any doses of other amines)
Non-heart-beating	Yes
**DCD**	
Age (yrs)	>50
BMI (kg/m^2^)	>35
Functional WIT (min)	>30
Macrosteatosis (%)	>30

*DCD, donation after cardiac death; DBD, donation after brain death; min, minutes; yrs, years; hrs, hours; CIT, cold ischemia time; HBV, hepatitis B virus; HCV, hepatitis C virus; BMI, body mass index; ICU, intensive care unit; AST, aspartate aminotransferase; ALT, Alanin-aminotransferase*.

Noteworthily, while livers from non-heart-beating donors are *per se* considered as ECD, Mihylov et al. suggested criteria for “ECD DCD” livers in order to subclassify DCD organs (26) (Table 2).

### ECD Pancreas

In analogy to other organs in SOT, no clear definition exists for a “marginal” or ECD pancreas graft. Table 3 summarizes the criteria that have been commonly used by various authors (27–30). In an attempt to bring such criteria into a clinically useful context, Axelrod et al. (31) presented a Pancreas Donor Risk Index (PDR). The score builds a prediction model of graft survival on donor factors together with ischemia time and type of transplantation (31).

**Table 3:** Common criteria defining a “marginal” or “ECD” pancreas.

Age (yrs)	<10/>45 (>50)
BMI (kg/m^2^)	>30
Trauma	Yes
Pancreatitis	Yes
Alcohol intake	Yes
DCD	Yes

*yrs, years; BMI, body mass index; DCD, donation after cardiac death; ECD, expanded criteria donor*.

### ECD Hearts

Due to the imminent gap between the supply of donor hearts and the demand for transplantable organs, strategies have emerged to overcome this clinical dilemma. The numbers of left ventricular assist devices as bridge-to-transplant therapy have significantly increased over the last decade. Simultaneously with this trend, marginal or “extended criteria” hearts are routinely evaluated in order to increase the donor pool. While standardized criteria have not been published by societies so far, there is a general consensus when it comes to the definition of marginal donor hearts. The literature on this topic is primarily driven by US data. A Meeting Report by the American Society on Transplantation on adult cardiac transplantation is still lacking a unified formal definition.

Based on the current literature, the factors that should be considered as extended criteria in heart donation are given in Table 4.

**Table 4:** Common criteria defining “marginal” or “ECD” hearts.

Age (yrs)	>40 (32)/>55 (33)
BMI mismatch donor/recipient (%)	>20
HCV	Positive
BMI (kg/m^2^)	79 (100.0)
LV hypertrophy (mm)	>14
Ejection fraction (%)	<45
High-dose catecholamine administration	Yes
Tobacco or illicit drug use (cocaine)	Yes
History of diabetes	
Prolonged cardiopulmonary resuscitation	Yes
Transient reversible hypotension or cardiac arrest	Yes

*LV, left ventricular; BMI, body mass index; HCV, hepatitis C virus*.

In order to facilitate a better risk-assessment of allografts, donor profiles have been implemented in Risk Scores. Smits et al. have identified 12 variables associated with donor non-use in the Eurotransplant region and have generated the Eurotransplant Heart Donor Score (32). Based on the UNOS database, a risk score model (UNOS Donor Risk Score) has been developed by Weiss et al. (33).

### ECD Lungs

Based on the standard criteria donor lung definitions published in 2003 by Orens et al. (34), one can define an ECD lung if the donor does not fulfill at least one criterion of the SCD criteria suggested by the International Society for Heart and Lung Transplantation (Table 5).

**Table 5:** Common criteria defining standard criteria (SCD), therefore not ECD lung.

Age (yrs)	<55
BMI mismatch donor/recipient (%)	>20
Clear chest X-ray	Yes
PaO_2_ (mm Hg)	>300 (FIO2 1.0, PEEP 5 mm Hg)
History of smoking (pack yrs)	<20
Absence of chest trauma	Yes
Absence of microbiologic organisms endobronchial	Yes
Absence of malignancy	Yes
Absence of purulent secretions or signs of aspiration endobronchial	Yes
Negative virology	Yes

*yrs, years; BMI, body mass index; FIO2, fraction of inspired oxygen; PaO2, partial pressure of oxygen; PEEP, positive end-expiratory pressure*.

## Machine Perfusion

The concept of MP was part of organ preservation long before solid organ transplantation (SOT) started to become a clinical reality and routine. With the publication by Collins et al., in which the authors described a method for the transportation of kidneys on ice using a preservation solution with the result that the organs could be shipped in a small box and showed almost no damage after 30 h, the era of static cold storage (SCS) had begun (35). The implementation of SCS led to satisfactory results within the entire field of SOT. However, with the increasing use of organs procured from ECD and DCD, SCS alone is not able to deliver the post-transplant results we aim to achieve for our patients. The lack of oxygen, the continuation of anaerobic metabolism leading to organ-damage and recipient-harming IRI after reperfusion, is significantly pronounced and more detrimental in these marginal donor organs (36).

**Figure 1:**
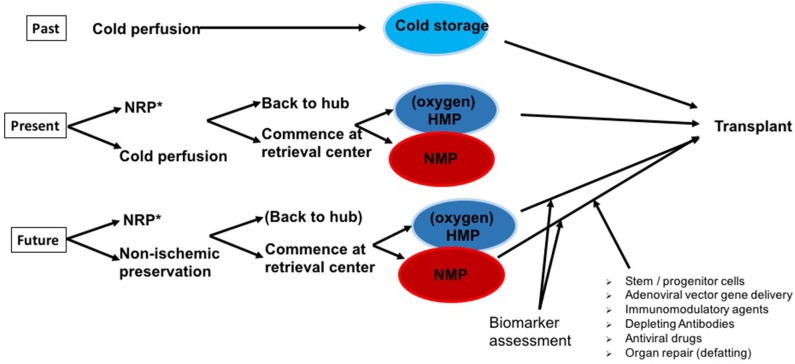
The past, present, and potential (near) future of organ preservation. Whereas, static cold storage (SCS) has been successfully applied over decades with good outcomes in standard criteria donor (SCD) organs, at present, marginal or expanded criteria donor (ECD) organs in particular are increasingly preserved by hypothermic (HMP) or normothermic machine perfusion (NMP) technologies. After initial cold perfusion [^*^and optimally following normothermic regional perfusion (NRP) in Donation after cardiac death (DCD) organs], machine perfusion is commenced either at the retrieval center or at the transplant center in a “back to hub” approach. The next step is to effectively use the prolonged preservation times of ECD grafts achieved by optimized machine perfusion protocols to characterize ECD organ quality and transplantability via the assessment of biomarkers. To improve graft quality, NMP provides an ideal platform for future immunomodulatory modifications and organ repair.

Figure 1 illustrates the different possibilities for combining all preservation techniques available today clinically for almost all organs: static cold storage (as the golden standard, so far), hypothermic preservation with/without oxygen, and normothermic perfusion. Normothermic regional perfusion (NRP) is mentioned in Figure 1, as it has become an important tool for use before the procurement process of DCD starts. The following sections will not present NRP data in particular, as the review is about machine preservation in marginal donor organs. However, good and satisfying results after kidney, pancreas, heart, and liver transplantation were reported by several groups in the United Kingdom, Spain, and France and have been summarized by previous reviews (37–42).

Recently, Ruiz et al. presented a series of 46 livers transplanted after NRP and concluded that their results, including 23% early allograft dysfunction, are superior to standard DCD organ procurements and comparable to donation after brain death (DBD) results (43). The Cambridge group (United Kingdom), clear NRP proponents, commented on this publication in a way similar to our suggestions illustrated in Figure 1. The publication by Ruiz et al. (43), like many others before it, did not compare the results to an adequate prospectively organized control group.

It is noteworthy that, only recently, a protocol for NRP was established even for DCD heart transplantation. Due to a restoration of function of the arrested heart, organ assessment via echocardiography, pressure-volume loops, and cardiac-output measurements could be implemented (44).

Accepted criteria for subsequent heart transplantation after weaning from mechanical support are defined as: cardiac index >2.5 L/min/m^2^; central venous pressure <12 mm Hg; pulmonary capillary wedge pressure <12 mm Hg; left ventricular ejection fraction > 50% in transoesophageal echocardiography.

NRP leads to a meaningful increment of survival benefits and helps to enlarge the available donor pool by utilizing marginal donor organs in a safe way (45, 46). Future trials are needed to compare DCD organ transplantation +/– NRP followed by +/– a combination of preservation techniques (42, 45).

Since 2009, dynamic cold storage—hypothermic machine perfusion (HMP)—has progressed to become clinical routine in several fields of SOT—above all, in kidney preservation. Moers et al. published their landmark paper in the *New England Journal of Medicine* and demonstrated a significantly lowered DGF rate in recipients receiving a hypothermically perfused kidney compared to a renal graft stored on ice in the common way (47). Applying the same technology (HMP without supplemental oxygenation), numerous publications on DCD and ECD kidneys have followed over the past 10 years, showing similar results: ECD and DCD kidneys and their recipients clearly benefit from MP as long as the duration of cold ischemia time (CIT) is reasonable (48–52). During the second half of the recent decade, oxygen as a supplement has been focused on for kidney HMP. In 2016, Jochmans et al. published an overview of ongoing MP trials in kidney and liver transplantation, including those assessing oxygenated HMP (53). One of these trials run by the COPE^®^ consortium (*http://cope-eu.com/work%20programme/trials.html**)* has finished recruiting, and 1-year-follow-up results were presented during the American Transplant Congress 2019. This international RCT on oxygenated HMP of DCD kidneys included 197 kidney pairs, of which 106 were successfully hypothermically perfused and transplanted. Approximately 80% of the transplanted kidney pairs were eligible for the primary analysis and resulted in a similar eGFR at 1 year after transplant for oxygenated (HMPO) and standard HMP. Analysis of all-cause graft failure, however, showed a higher eGFR in HMPO than in HMP. Overall graft loss was significantly lower in HMPO, leading the authors to suggest that HMPO improves 1-year kidney graft function when accounting for the beneficial effect on allograft survival.

In contrast, in the field of pancreas transplantation, dynamic preservation technologies have not yet experienced a breakthrough. Neither hypothermic nor NMP technologies are well-established, and SCS remains the standard procedure. A recent review outlined nine studies on HMP and 10 studies on NMP. All of them were experimental; none of the human pancreatic grafts were transplanted. However, the common conclusion of all of the published articles considered was that IRI, thrombosis, and morbidity after whole organ pancreas transplantation might be reduced by both technologies (54–56).

Liver transplantation can be regarded as the field in SOT that primarily focuses on the development of preservation techniques, currently. The most commonly used perfusion types in the daily routine are HOPE (hypothermic oxygenated perfusion), D-HOPE (d for dual oxygenation via hepatic artery and portal vein), and normothermic liver preservation. HOPE and D-HOPE studies have produced promising results, showing higher adenosine triphosphate (ATP) levels during preservation, less IRI, excellent graft survival after DCD liver transplantation, and a significantly reduced rate of bile duct injuries (57–60). The Birmingham group was the first to publish its experiences on livers undergoing HOPE and NMP consecutively (61). Although such livers were not transplanted, HOPE/NMP livers developed less oxidative injury and inflammation and achieved enhanced metabolic recovery compared to livers undergoing NMP only (61). Liver NMP had its great appearance when the first prospective international multicenter RCT was published in April 2018 (62). The trial on DBD and DCD livers with NMP durations up to 24 h could show a significantly lower level of graft injury, measured by reduced peak aspartate aminotransferase (AST), longer preservation times, and a 50% lower discard rate of organs compared to SCS. Bile duct complications and graft and patient survival were comparable in both groups (62).

NMP has been shown to be beneficial in terms of immediate graft function in all organ types in which it has been explored clinically: this includes the kidney (63–65), liver (62, 66), and the lung (67, 68). The mechanism by which this occurs has not yet been conclusively proven, but the concept is that reperfusion (rewarming and oxygen delivery) in the artificial context of the perfusion circuit is beneficial by allowing recovery of cellular energetics in the absence of the effector mechanisms needed for an acute inflammatory response (as in ischemia-reperfusion).

## Organ Preservation vs. Organ Conditioning vs. Organ Repair

With a new technology, the accompanying terminology is often somewhat unspecific and unprecise in the beginning. This also applies to MP. It is paramount, however, to eventually clarify the terminology and define a uniformity for its correct use. Simply put, no other terminology than organ preservation would be justified at this point in the clinical application, since no targeted means for organ reconditioning (let alone repair) have been established. While this remains a glorious goal for the future, it also represents a significant legal and ethical challenge, since the framework for the treatment of human organs remains to be established. It remains entirely unclear whether extracorporal organ reconditioning and/or repair can be achieved. The hope and the hype are building on a growing body of experimental data that indicates the feasibility of success or organ-specific treatment. While this remains a most significant opportunity for the field, we herein aim to adequately define organ preservation, organ (re-)conditioning, and organ treatment.

### Organ Preservation—A Definition

It is interesting to note that no entry exists on organ preservation in Wikipedia (search, 21.08.2019), while a PubMed search yields 17189 entries (*https://www.ncbi.nlm.nih.gov/pubmed/?term=organ+preservation**)*. It seems to be a term where the definition appears so clear and easy that it does not need to be written down. Yet again, a uniform description of organ preservation is not straightforward since it is not necessarily defined by organ retrieval, temperature, metabolic state, or any other sole condition. The one possible common ground for a definition is the purpose. Putting an organ into a state that allows later reactivation and restitution of its original function with the aim of minimizing damage during the period of mal- and non-perfusion.

One essential limiting factor in the determination of the quality of the preservation method is the poor definition of endpoint for a clinical readout. Primary non-function (PNF) seems relatively well-defined at first glance, but the actual definitions leave room for interpretation. PNF does not indicate no organ function at all but rather a function insufficient to prevent/avoid longer-term organ replacement therapy.

For example, in liver transplantation, according to Ploeg et al., PNF is defined by poor initial function, requiring re-transplantation or leading to death within 7 days after the primary procedure without any identifiable cause of graft failure (69). Such parameters inevitably introduce non-specificity into the definition. The call for the need for re-transplantation and the risk for death are clinician's calls built on medical facts, the interpretation of the physician, and a projection based on the parameters and the experience of the doctor. Since alternatives are insufficiently well-established, studies relating preservation quality to PNF need to be read in the light of this limitation. While PNF is seen as a suitable measure for organ preservation, other factors impacting on the PNF rate need to be considered. The fact that re-transplantation is a risk factor for PNF indicates that the role of recipient factors may be underestimated (70). Further to this, no clinical preservation trial can seriously build on PNF as an endpoint, since the PNF rates reported more recently are in the range of 2–5%. Primary poor function is even more vague in its sufficiency as a definition and clinical endpoint. Very recently, Dutkowski et al. (71) critically reflected on the current parameters used to determine the success of preservation in liver transplantation trials. While the current practice might be reasonable in the sense that it is building on the combination of parameters currently best established, alternatives are needed but need to be more formally established prior to considering them new clinical standards.

### A Brief History

The original vision of organ preservation as formulated in 1813 was built on the continuation of circulation and blood supply (72). The substitution of blood with perfusion solutions led to prolonged successful preservation of tissues, as described by Carrell (73). While prolonged successful preservation of tissues seems plausible according to these early findings, the actual suitability for transplantation remained to be established (72). Successful kidney preservation for 3 to 5 days was achieved by the use of continuous perfusion with cooled, oxygenated blood or plasma (74). Since continuous warm perfusion, oxygenation, and nutrition of an organ during transportation was technically very demanding, the alternative concept eventually surpassed MP in the 1960s. The chemical composition of advanced solutions for hypothermic organ storage resulted in excellent short-term preservation with good outcomes after transplantation. As with any achievement, the good results are a blessing and a curse. With patient and graft survival as the only hard endpoints for clinical trials, the room for improvement became very small—at least with standard criteria organs. The search for superior technologies found a platform for clinical realization only when the results after transplantation became less convincing, with a greater effect on bile ducts as a target for early damage in liver transplantation (72, 75–77). The organ shortage, together with the push to use lower-quality organs and organs with additional damage as a result of DCD, triggered the clinical realization of MP in liver transplantation.

### Current Situation

The current status of organ preservation remains largely defined by SCS. The well-established and standardized procedures of organ storage in three sterile plastic bags filled with preservation solution (bag 1) or saline/ringer lactate/other (bags 2/3) is well-established, with a relatively good understanding of the risk accumulating with the duration of preservation. The major advantage of SCS is the safety of the procedure. Pretty much nothing can happen to an organ in plastic bags, on ice, in a plastic container. The major limitations of this technique relate to the fact that metabolism continues—even though at a very low rate—eventually resulting in the accumulation of succinate and reactive oxygen species (ROS) (78–81). The immediate exposure to normothermia and oxygen results in an inflammatory response and organ-specific damage early after transplantation. The mechanisms responsible for the eventual organ damage not only in organ transplantation but also in other diseases such as cardiac infarction and stroke are well-established but not are the focus of this review. The key question in the consideration of NMP as an alternative to SCS is how effectively the mechanisms leading to an IRI are interrupted through either immediate NMP or NMP following SCS. While preclinical evidence (81) and the phase II/III trial by Nasralla et al. (62) have delivered evidence suggesting that NMP is superior to SCS when applied immediately, the actual net benefit of NMP following SCS remains to be more clearly established. While a key elements in such assessments are the proper definition of and agreement in the community on suitable endpoints regarding the preservation quality, the accompanying benefits and alternative endpoints should not be overlooked. The major benefit of NMP vs. SCS is the time gained with (as it appears) a relatively safe prolongation of preservation times under close to physiological conditions. The ability to monitor the viability and function of the organ adds important parameters for decision making and significantly ameliorates the logistical challenges in transplantation. The benefit of transforming transplantations into scheduled procedures has the potential to significantly enhance safety but also training and teaching in transplantation.

### Organ Reconditioning

The term organ reconditioning is used in a rather unspecific manner in our field. According to Wikipedia, reconditioning means “to restore to a functional state, or to a condition resembling the original” (*search “reconditioning,” September 2019*). The way this can be read in reference to human organs is that no intervention aiming beyond the restoration of the condition of an organ prior to retrieval (a) or beyond the optimal (naïve) condition of a healthy human organ (b)—depending on how the term “original” is interpreted in this context—shall be included. These two possible views already point toward very different states of an organ. The way the term was used in the recent scientific transplant literature is in agreement with repair of the immediate injury prior to and during organ retrieval and storage. The key element of reconditioning/repair as clinically applied at this point relates to the prevention of an eventual ROS burst by mitochondria and subsequent disruption of ATP production, mitochondrial permeability transition pores, and danger-associated molecular pattern (DAMP) release upon reperfusion. An electron shift toward succinate and subsequent reverse electron transfer upon reperfusion is discussed as a mechanism of damage and target of therapy in liver HMP (82). NMP is suggested to mitigate post-reperfusion hyperfibrinolysis (83) and inflammation (84). While these may be indirect effects of NMP, the actual immediate therapeutic mechanisms (if any) may be glycogen repletion and ATP regeneration (85). Whether these mechanisms are a simple effect of the restoration of close to physiological conditions or specific for NMP remains to be established. It is likely, however, that the benefit of NMP is mostly defined by the replacement or shortening of SCS and the indirect effects on logistics and preparation, organ monitoring, and decision making. Further to this, NMP may emerge as a platform enabling organ treatment, while it may not have a therapeutic/reconditioning effect *per se*.

### Organ Repair

Human organ repair, apparently, is treated as an equivalent to organ regeneration (*Google search “human organ repair,” September 2019*). While the common understanding of organ repair might be more mechanistic and that of organ regeneration more biological, the distinction between the terms seems vague.

Albeit experimental, therapeutic interventions in lungs seem most closely approaching possible clinical implementation (86). Bronchoalveolar lavage, surfactant replacement, and alveolar recruitment maneuvers seem to positively affect organ tissue morphology and organ function. A hurdle to clinical application may be the legislation. Directive 2004/23/EC of the European Parliament and of the Council “on setting standards of quality and safety for the donation, procurement, testing, processing, preservation, storage, and distribution of human tissues and cells” largely focusses on tissues and not organs but represents an important reference. Since preservation times can be prolonged with the technologies being developed and urgency may no longer serve as justification for immediate processing (transplantation) without quality assurance, the legal framework for tissues and cells could be applied to organs. The second element of the legal consideration relates to the element of organ therapy. Inevitably, the issues of responsibilities and possession require clarification for attempts to introduce *ex vivo* human organ treatment into clinical application. As long as an organ is labeled as discarded because it seems unsuitable for immediate transplantation, the organ will not be allocated to an individual, and hence considerations of possession and responsibilities become less demanding. Once organs possibly suitable for immediate transplantation have been treated for reasons such as quality improvement or possibly organ reconditioning, the allocation process may be completed and the future recipient indicated. If and how an individual can consent to interventions and/or a trial involving organs will eventually require clarification.

Directive 2010/45/EU of the European Parliament and of the Council of 7 July 2010 on standards of quality and safety of human organs intended for transplantation (*https://eur-lex.europa.eu/eli/dir/2010/53/oj**)* does not provide much guidance regarding this issue.

## Machine Perfusion in Clinical Practice

SCS is a simple, safe, and cheap method to preserve and transport organs, and it has been successfully applied over decades with good outcomes in SCD organs. However, to date, the large discrepancy between patients on the waiting lists and available donor organs has forced the transplant community to cope with the demand by pushing the limits and expanding the donor pool by accepting increasing numbers of marginal organs from ECD and reestablish DCD programs (87). Importantly, ECD and DCD organs have been identified to be particularly prone to IRI. Although SCS can decelerate degradation of a preserved organ, especially in high risk or marginal organs, limited applicability is the consequence (36, 78). Attempting to overcome this issue, several MP technologies have been developed and now find their way into clinical practice in SOT.

### HMP of the Liver

Liver HMP has been translated from the experimental state to clinical reality over the last decade. In 2010, the first phase I prospective cohort study was published, reporting on 20 patients who underwent liver transplantation of grafts preserved by non-oxygenated HMP. Outcomes were compared to a matched SCS control group. HMP time ranged from 2 to 7 h, with a total CIT below 12 h. Except for a significantly lower peak serum AST in the HMP group, no further significant differences were observed in regard to PNF, early allograft dysfunction, or graft and patient survival. However, clinical feasibility, as well as non-inferiority to SCS, was proven, making way for further studies (88). Five years thereafter, the same group showed promising results by transplanting 31 ECD livers declined by the United Network for Organ Sharing (UNOS) region and preserved under hypothermic dynamic conditions at 4–8°C. Compared to well-matched SCS controls, significantly fewer biliary complications and shorter hospital stays were observed. This study represents a landmark, since the effectiveness of MP was shown within the most susceptible livers in the donor pool (89). The impact of HMP on marginal organs was further investigated by Dutkowski et al. In an international matched-case analysis, 25 DCD livers were subjected to HOPE and compared to matched SCS DCD livers. HOPE resulted in a significant reduction of ischemic cholangiopathy (HOPE: 20% vs. SCS: 46%) as well as a significant improvement of 1-year graft survival rates (90 vs. 69%) (58). The beneficial effect of HMP on DCD grafts was confirmed by a prospective case-control liver transplantation study, comparing 10 grafts with at least 2 h of DHOPE to 20 grafts without. An 11-fold increase in cellular ATP during oxygenation was documented. In addition to good early graft function, both 6-month and 1-year graft survival were 100%, while 6-month graft survival and 1-year graft and patient survival in the control group were 80, 67, and 85%, respectively (57). Moreover, the same group could demonstrate an attenuation of IRI-associated bile duct damage after transplantation of DCD end-ischemic DHOPE liver grafts (90).

HOPE in particular has evolved to become a widely used technology, showing excellent longer-term results. Schlegel et al. provided data on 5-year outcomes of patients receiving DCD livers preserved with 1–2 h of end-ischemic HOPE (*n* = 50) and compared them to the equal numbers of DCD liver transplants without HOPE or DBD liver transplantations. Five-year outcomes of HOPE-treated DCD liver transplants were similar to those of DBD primary transplants and superior to those of untreated DCD liver transplants (HOPE DCD 94% vs. SCS DCD 78%) (91).

### NMP of the Liver

Preserving an organ under physiological conditions is the ideal condition to prevent deleterious effects of ischemia as well as IRI following SOT. Similar to HMP, NMP has recently been implemented in clinical routine at a variety of centers. The transfer from the experimental stage to clinical application was first described by Ravikumar et al. in 2016. In this phase 1 (first-in-man), non-randomized prospective trial, short-term outcomes of 20 recipients of NMP-perfused donor livers were matched in a ratio of 1:2 to cold-stored livers. In the study group, livers were preserved under normothermic conditions over the entire preservation period (ranging from 3.5 to 18.5 h). Except for a significantly reduced peak serum AST in NMP-liver recipients, no differences regarding short-term recipient survival, PNF rate, or early allograft dysfunction were observed. Therefore, the safety and feasibility of this dynamic preservation method could be confirmed, opening the field for further clinical investigations with major implications on logistics (66).

Feasibility and reproducibility following transplantation were further underlined in a U.S. phase I non-randomized pilot study (92). However, this trial also pointed out that NMP of an organ is not a trivial procedure and runs the risk of graft loss if human/technical errors occur. Out of 10 procured livers, one organ had to be promptly discarded due to an unrecognized portal vein twist. The remaining nine organs (four DCD Maastricht class III, 5 DBD) were successfully transplanted, and outcomes were comparable to matched control liver transplant recipients of SCS grafts. The proof of concept and feasibility gave way for larger-scale trials.

Nasralla et al. were first to perform RCT to test the efficacy of NMP vs. SCS. After informed consent had been obtained from the recipient, the allocated liver was either randomized to SCS or NMP. NMP was performed over the entire preservation period. Outcomes of 121 NMP-graft recipients were compared to outcomes from 101 well-matched SCS-graft recipients. The key findings of this study were a significantly reduced peak serum AST, a significant reduction of post-reperfusion syndrome, and a 72% lower adjusted odds rate of developing early allograft dysfunction (EAD) in the NMP liver recipients compared to controls. Furthermore, NMP of the liver graft resulted in increased organ utilization with a 50% lower discard rate in this group (62).

The additional positive effect of NMP is the “almost” physiological aspect of this method, which opens up new possibilities by means of organ assessment and organ selection. This fact was a matter of investigation in an observational study, where livers considered unsuitable for transplantation as well as highest risk liver were included. In total, 47 livers were biochemically assessed during the preservation, resulting in 39 liver transplants. Remarkably, two out of 19 livers deemed unsuitable were transplanted (93).

In summary, both HMP/HOPE and NMP are accepted and clinically established preservation methods, particularly interesting in the preservation and preconditioning of high-risk organs. Especially in this organ category, including ECD and DCD livers, they have both been shown to be superior to SCS. Whether HMP/HOPE or HMP is superior to the other is still a matter of debate since the two methods have not been correlated with each other in clinical settings, and further RCTs are needed.

### HMP of the Kidney

The amount of clinical evidence for dynamic preservation of kidney grafts is rapidly increasing. Moers et al. were first to perform an international RCT on hypothermically perfused kidneys for transplantation. One kidney from 336 deceased donors was randomly assigned to HMP, and the other to SCS. A reduced risk of DGF and improved graft survival in favor of MP-preserved organs was shown (47). These promising results were confirmed in a subsequent RCT from the Eurotransplant region. Jochmans et al. investigated the efficacy of HMP in preserving DCD kidneys. Similar to Moers et al., DCD kidneys were assigned in a 1:1 match to HMP or SCS, resulting in a total of 164 kidney transplants (82 HMP vs. 82 SCS). HMP resulted in a significantly reduced DGF rate as well as better early graft function after 1 month compared to SCS. However, 1-year graft survival was comparable in both groups (49).

Furthermore, in another RCT, kidneys were either preserved by HMP or SCS. In accordance with Moers as well as Jochmans, the primary end-point was the occurrence of DGF. Secondary endpoints were primary non-function as well as graft survival. Both the risk of DGF and the incidence of non-function were significantly reduced by HMP in their cohort. One-year graft survival and function could be improved by dynamic cold graft preservation (52). Despite these findings, the importance of different factors such as the impact of CIT and the combination of CIT with HMP have to be mentioned. CIT is a known independent risk factor for DGF. In order to investigate which kidney graft may further benefit from HMP prior to transplantation, Kox et al. analyzed prospectively collected data from the “Machine Perfusion Trial.” A total of 752 renal transplants were included with 376 dynamically preserved kidneys and 376 statically preserved grafts. They identified CIT as an independent risk factor for DGF. Furthermore, HMP did not prevent DGF if CIT exceeded 10 h (51).

The optimal timepoint for, as well as the optimal duration of, HMP is still a matter of debate. HMP can be used either as a primary preservation method or in the end-ischemic phase as a reconditioning tool. Matos et al. were able to show a faster recovery of renal function if grafts were subjected to HMP prior to transplantation after a mean SCS period of 22 h. MP time was at least 6 h. However, this beneficial effect was limited to donors under the age of 50, who represent a rather selective group (94). Further studies followed, indicating a beneficial effect of MP in the end-ischemic phase by displaying a reduction of DGF, as well as an improved organ acceptance. This last positive trend is attributed mainly to the possibility of organ monitoring (95, 96).

### NMP of the Kidney

NMP of the kidney may eventually resolve many issues related to damage induced by ischemic phases. The idea of preserving kidney grafts under almost physiological conditions with the possibility of real-life assessment of the graft prior to transplantation sounds intriguing. However, while the number of preclinical studies using animal models as well as discarded human kidneys is steadily increasing, evidence for NMP of the kidney in a clinical setting is rather poor. Hosgood et al. were first to transform preclinical experience into clinical reality by transplanting a 62-year-old ECD kidney into a 55-year old recipient. After 11 h of SCS, the graft was perfused with a plasma-free red cell-based solution at 33.9°C for 35 min prior to transplantation. Although a slow graft function was observed, the patient remained dialysis free with a serum creatinine level of 132 μmol/L at 3 months post-transplantation. In contrast, the recipient of the opposite kidney developed DGF (64). Based on this first success, the Leicester group translated NMP into clinical reality on a larger scale. Eighteen ECD kidneys were subjected to NMP reconditioning prior to transplantation. The average NMP time was prolonged to 63 min. Outcomes were compared to 47 matched ECD kidneys preserved by SCS. In this study, NMP resulted in a significantly reduced DGF rate compared to controls (NMP: 5.6% vs. SCS: 36.2%). However, no differences could be observed in regard to 1-year graft or patient survival (97).

Taken together, HMP of the kidney has been shown to have its place, especially in the preservation of marginal grafts, including grafts from DCD and ECD, with superior outcomes in the early phase compared to SCS. However, CIT is still the limiting factor influencing short- as well as long-term outcomes. In contrast, although the amount of preclinical data is rapidly increasing, NMP is still in its early phase. However, feasibility and non-inferiority to SCS have been described at this early stage, and multicenter RCTs are planned.

### Machine Preservation of the Pancreas

In contrast to the dynamic preservation of liver and kidney, pancreatic MP is challenging. This is attributed to the physiologic as well as the anatomical characteristics of the pancreatic graft (98). MP of pancreatic grafts poses major challenges due to the susceptibility of the organ to IRI-associated alterations, including acinar necrosis, edema formation, and endothelial disruption. These factors in particular are recognized risk factors for early graft pancreatitis and thrombosis, eventually resulting in graft loss (98, 99).

In contrast to the liver as well as the kidney, the pancreas is characterized by a low-flow and low-pressure environment, which impedes the direct translation of the existing perfusion machines for use in dynamic pancreas preservation (100). However, the feasibility of pancreatic HMP has been demonstrated in several experimental settings involving the preservation of porcine, dog, and discarded human pancreas grafts (55, 101, 102). Although results are promising in this experimental setting, clinical translation is still pending.

Therefore, assessment and evaluation of a pancreatic graft prior to transplantation would be of the utmost importance, since changes in donor demographics result in an increased need for the acceptance of higher-risk grafts at high-volume centers. However, MP of pancreatic grafts must still be considered experimental at this stage.

### Machine Preservation of the Lung

Due to pre- and peri-retrieval management and direct examination of the donor organ by the explant surgeon, some extended criteria organs can be directly used with acceptable risk for the recipient, but a notable number are rejected during the retrieval process. There is still a lack of clear decision criteria for lung suitability for transplantation or attempts at lung reconditioning using an *ex vivo* lung perfusion (EVLP) (103). Needless to say, MP could improve organ availability, which is critically needed in the face of a waitlist mortality that is reported to be up to 30% depending on the allocation system (104).

Importantly, *ex vivo* prolonged lung preservation resulted in successful transplantation of high-risk donor lungs (105), and MP is now indispensable for evaluating lung graft quality from an uncontrolled DCD (106). Very promising data have come from experimental EVLP studies, which are outlined later in this article.

### Machine Preservation of the Heart

At present, SCS of donor hearts remains the standard practice in most transplant units, whereas MP is limited to a few centers in the US and Europe. To date, only one device is available for clinical use—the Transmedic Organ Care System (OCS) Heart. At mild hypothermia (34°C), the system uses a combination of donor blood and a proprietary solution as perfusate for the heart. In 2014, heart NMP has led to the first distant procurement DCD heart transplantation. To date, over 100 DCD heart transplants have been performed using a cardiac perfusion system (107), and MP of hearts exhibited safe preservation in RCTs (108).

## *Ex vivo* Monitoring of Organ Function and Quality

Currently, the decision as to whether organs are suitable for transplantation is determined on the basis of more or less subjective empirically established clinical parameters that have been shown to be associated with an increased rate of early allograft dysfunction or graft failure. Parameters such as donor past medical history, last known laboratory values, findings during procurement, and other procurement variables such as expected ischemia times primarily determine the acceptability of a graft. These parameters include prolonged warm ischemia time (WIT) >30 min during DCD, prolonged CIT, and parenchymal alterations within the graft (e.g., steatosis, fibrosis, arteriosclerosis). Viability testing and functional assessment prior to transplantation are likely to extend the utilization of suboptimal or marginal organs (109–111).

HMP is an alternative method to standard SCS for the preservation of organs. For different types of kidney grafts, HMP offers superior preservation compared with SCS. In terms of graft quality assessment during HMP, some biochemical parameters of the released perfusate and hydrodynamic parameters are found to independently correlate with the outcome—a finding that may help clinicians in their decision making.

Increased Glutathione S-Tranferase (GST), N-acetyl-beta-D-glucosaminidase (NAG), heart-type fatty acid-binding protein (H-FABP), or lactate dehydrogenase (LDH) concentrations during MP may serve as indicators for suboptimal graft quality. Furthermore, levels of redox-active iron measured in the perfusate have been correlated with DGF rates (112–114).

Moreover, the measurement of vascular resistance during MP represents an additional and objective source of information that can assist clinicians in their decision-making process. High vascular resistance as a hydrodynamic parameter has been shown to be associated with an increased risk of DGF development and predictive for 1-year graft failure (115–117). Taken together, to date, increased vascular resistance and high injury marker concentrations in the perfusate are risk factors for DGF and helpful parameters, but they are not accurate enough to justify a decision for discard based on their interpretation alone (112–114, 117).

Concerning kidney NMP, a scoring system has been developed by Hosgood and Nicholson. The decision on whether to transplant an NMP-kidney was based on macroscopic appearance, renal blood flow, and total urine output during NMP. The authors conclude that, currently, a high percentage of retrieved kidneys are being unnecessarily discarded (80, 118).

Compared to the kidney, data on liver monitoring during HMP remain scarce, although recent publications suggest promising parameters. A correlation between the AST levels in the perfusate during HMP and the peak of AST after liver transplantation has been described. Furthermore, during HMP of porcine livers, a cumulative release of Fatty Acid- Binding Protein 1 (FABP) or AST may be represented by a linear or logarithmic equation, respectively. Each equation is characterized by a b-coefficient that is able to discriminate between livers likely to fail. Similarly, during HMP of human livers discarded for transplantation, AST release in the perfusate could discriminate between livers suitable for transplantation and unsuitable ones. More sophisticated methods to determine graft viability include evaluation of ATP content assessed by magnetic resonance imaging and spectroscopy (89, 91, 119–121). In this regard, van Rijn et al. used cellular ATP content during oxygenated dual (arterial and portal perfusion) HMP as a viability marker for liver grafts. Cellular ATP content correlated with biochemical function early after transplantation (57).

Furthermore, there is evidence that a decline in arterial flow in a pressure-controlled system of MP can be used as a marker for decreasing graft viability. However, in general, it cannot be advised to use flow values as an indicator of liver damage and viability during human liver MP (61, 91, 122).

Concerning NMP of liver grafts, this technology has been applied with promising results for utilization and outcomes (62, 66, 92, 123–126), and the option for the assessment of liver viability during NMP has been highlighted. Imber et al. (127) suggested that bile production is directly associated with liver viability. Matton et al. (128) were able to show that a biliary bicarbonate concentration greater than 18 mmol/L has a high negative predictive value in terms of histological bile duct injury. Moreover, biliary pH greater than 7.48, biliary glucose concentration less than 16 mmol/L, bile/perfusate glucose concentration ratio less than 0.67, and biliary LDH concentration less than 3,689 U/L may serve as indicators for high biliary viability. Watson et al. (93) published their experience of transplanting declined livers following NMP and graft viability assessment. They observed that NMP resulted in a reduced incidence of post-reperfusion syndrome and described biliary pH as a predictive marker for post-transplant cholangiopathy. Perfusate lactate and transaminases and bile production during NMP were suggested by Op den Dries et al. (129) to serve as viability markers. Recently, Mergental et al. (130) published viability criteria (applicable within 3 h of perfusion) for livers considered suitable for transplantation. These include lactate clearance, bile production, PH- homeostasis, and stable pressure and flow dynamics of the graft.

There is growing evidence that hepatic ATP content may serve as a viability marker. In clinical studies, hepatic ATP levels prior to organ retrieval or during preservation correlated with primary liver function after transplantation. Bruisma et al. showed that differences in mitochondrial respiration and restoration of cellular ATP contents are possible viability markers in the setting of NMP. Moreover, NMP offers the option to perform dynamic metabolomic analyses in both bile samples and sequential liver biopsies (111, 131).

However, although these results seem impressive, none of the above-mentioned viability markers have been validated in larger studies, and the small numbers of patients included in the studies make it difficult to draw robust conclusions.

Regarding a potential viability assessment of human pancreas grafts during HMP, flow indices and histological assessment of duodenal and pancreas-parenchyma biopsies throughout the perfusion duration have been proposed. Branchereau et al. described an absence of edema, a decrease in resistance indices, and normal staining for insulin, glucagon, and somatostatin as markers for good graft quality (56, 101). Barlow et al. suggested the assessment of amylase levels in the perfusate, fat infiltration (detected using standard histology), and exocrine pancreas function as criteria to assess graft viability during NMP (55).

Concerning heart transplantation, organ assessment during NMP mainly relies on metabolic parameters and measurements of lactate levels in the aortic root and pulmonary artery. In the PROCEED II trial, three hearts were discarded due to rising total perfusate lactate concentrations. Histopathological examination showed myocardial injury (necrosis, hemorrhage, scarring) or left ventricular hypertrophy in those organs (108). Calculations of pressure volume loops in the left and right ventricle have been described by switching the heart into a “working mode” during NMP. Based on pressure-volume (PV) loop data, end-systolic elastance could be calculated as a parameter for ventricular contractility (44, 132). Due to complicated clinical interpretation and difficulties in reproducibility, “working mode” has been removed from the OCS perfusion module. Contrast echocardiography or intravascular ultrasound of coronary arteries might emerge as tools for anatomical and mechanical assessment during NMP but have not been implemented in clinical routine so far.

In EVLP, assessment of the organ relies primarily on functional and macroscopic parameters, as has been reviewed recently (103). Adequate gas exchange, stable hemodynamic and ventilatory parameters, macroscopic evaluation (absence of oedema), bronchoscopy (absence of purulent secretion and erythema of the bronchus), and deflation after endotracheal tube disconnection (collapse test) during the evaluation period are the commonly used decision criteria for acceptance of the lung. Yet these criteria are not standardized and differ between transplant centers (103). As physiological parameters are in the lead for decision making in EVLP, clinical single-center studies assessed different biomarkers and metabolic parameters of the perfusate or the bronchioalveolar lavage to predict organ acceptance and occurrence of early prostaglandin D3 (PGD3). Machuca et al. (133) showed that high levels of endothelin-1 (ET-1) and Big ET-1 measured in perfusate samples have prognostic value for decline in the lung because of subsequent development of poor physiological performance. Subsequently, the same group suggested IL-8 as a powerful predictor for early graft dysfunction (134). Furthermore, IL-1β and IL-8 levels measured in the perfusate during EVLP were reported to inversely correlate with the recipient oxygenation 24 h post-transplantation (135).

Interestingly, given the observation that perfusate IL-1β and TNF-α at 30 min during EVLP were effective markers for differentiating between in-hospital survival and non-survival post-transplant, blocking the IL-1β pathway during EVLP might reduce endothelial activation and subsequent neutrophil adhesion on reperfusion (136).

## Immunological Aspects of (Marginal) Organs During Machine Perfusion

The definition of marginal organs *per se* is a rather arbitrary one. The most commonly used is the dichotomy between standard criteria (SCD) and extended criteria donors (ECD), which is simply discriminated by age and three donor criteria for the DBD (and, increasingly, DCD) kidney cohort (13, 137, 138). However, in clinical routine, it is not always straightforward to decide if an organ is within “standard criteria,” as there are plethora of factors interfering with organ quality, i.e., estimated duration of CIT, (functional) WIT, or the grade of steatosis in liver and pancreas, just to mention a few.

When we focus on the donor age only, a vast amount of literature exists. There is evidence that the immunogenicity of elderly organs is increased, as it is believed that aging in combination with injuries (i.e., brain death) induces a pro-inflammatory environment, which could lead to an activation of innate and adaptive immune responses (139–141). Aging influences cellular repair mechanisms, and it is known that the number of MHC molecules present on the cellular surface is increased by aged parenchymal cells (139). In experimental studies, transplantation of elderly organs was associated with a more powerful early immune response compared to transplantation of younger ones. Recipients of such old grafts presented with a higher concentration of effector/memory T-cells with an increased alloreactivity, leading to acute rejection, which results in a particular high burden of damage for the older donor organ (140). Elderly organs do not have the same repair mechanism capabilities, so the clinical damage of acute rejection leaves a more severe defect, explaining the increased rates of DGF and/or graft loss in this donor cohort (140, 142).

An alteration of immunogenicity in aging organs could be one reason to focus on preservation techniques (143) such as HMP and NMP to learn more about the process or even to (re-)condition an organ for reperfusion in the recipient. Due to the fact that the reconstitution of a near-physiological environment might work best to study immune cells, the following paragraphs will bring NMP into focus.

Human organs are equipped with a sophisticated resident immune system. The kidney, for example, not only hosts tissue-resident macrophages (144); the organ also harbors lymphocytes, innate lymphoid cells, natural killer (NK) cells, natural killer T (NKT) cells, and γδ T cells (145). The residential immune cells of human organs remain in a balanced homeostatic state unless they are stimulated—by the insult of brain death in an organ donor, for example—when an immunological activation might be induced. Such a response to a significant injury like brain death in an organ donor includes both local and systemic inflammation, comprising cellular infiltrates and a cytokine storm (146). With the current gold-standard preservation technologies such as SCS, the organs will be transplanted unchanged, in an immunologically activated status, into recipients in whom alloantigens will trigger another immune response immediately after reperfusion, presented by donor-derived antigen presenting cells to recipient T cells (147, 148). How important donor-derived leukocytes are in the allorecognition and rejection processes was demonstrated by Lechler and Batchelor years ago. They impressively showed that placing a rat kidney into an intermediate recipient before implanting it into the final recipient could significantly prolong allograft survival. These experiments demonstrated that “clearing” the organ of dendritic cells (DC), the “passenger leukocytes” responsible for activating recipient T cells, by using the intermediate rat led to the desired success (149, 150). “Parking” the organ in an interim host is not a feasible strategy for implementation in the clinical routine. However, NMP offers the possibility to represent the intermediate recipient as a conditioning tool to achieve the same end result, as previously published by Lechler et al. (150). An *ex vivo* setting might provide not only a more physiological preservation method but also the ability to assess the organ prior to transplantation and to investigate its immunological characteristics in isolation (42).

One of the leading groups analyzing marginal and SCD organs after *ex vivo* perfusion is the Manchester group around Fildes and Stone. Already in 2015, they compared the clinical outcome of patients transplanted with marginal donor lungs after undergoing EVLP compared to standard transplantation of acceptable lungs (151). Despite having transplanted marginal lungs, there was no significant difference in the clinical outcome up to 12 months after transplantation concerning the overall incidence of acute rejection and the number of treated infection episodes (151). This work was followed by a study on passenger leukocyte migration from donor lungs into the recipients and the effects of donor leukocyte depletion prior to transplantation using EVLP (152). In this experimental work using a porcine model, Stone et al. illustrated that donor leukocyte transfer into the recipient was reduced by EVLP and therefore reduces direct allorecognition and T-cell priming (152). The same group transferred their experience to an *ex vivo* NMP model to analyze donor-derived leukocytes in a porcine renal transplantation model. Stone et al. were able to demonstrate that an inflammatory cytokine storm and the release of mitochondrial and genomic DNA were initiated in the NMP circuit prior to transplantation. However, the renal function was not impacted at all after transplanting those organs. They suggest that NMP could be used to immunodeplete and to saturate the capacity of inflammation of donor kidneys before transplanting them (152). Amin et al. (153) evaluated the impact of a post-SCS-preservation flush on the inflammatory burden of a limb allograft (a porcine model for vascularized composite allotransplantation). The venous effluent after flushing the limbs following either 2 or 6 h of SCS comprised a large population of viable leukocytes, significant concentrations of pro-inflammatory cytokines and mitochondrial DNA. These results support the hypothesis that flushing or dynamically preserving the organs prior to transplantation impacts the inflammatory burden otherwise transferred to the recipient upon reperfusion unchanged (153).

Liver NMP has recently been implemented as a standard procedure in the United Kingdom and been shown to improve organ utilization and post-transplant outcomes following phase I and phase III prospective randomized trials (62, 66). Jassem et al. (84) transplanted, compared, and analyzed 12 NMP-preserved livers against 27 SCS-preserved livers to assess the impact of NMP on IRI, necrosis, platelet deposition, neutrophil infiltration, and the degree of steatosis after NMP or SCS pre- and post-perfusion. Their results showed that, with NMP, there were altered gene-expression profiles of liver tissue from pro-inflammation to regeneration, reduced numbers of interferon gamma (IFNγ)- and interleukin-17-producing T cells, and an enlarged pool of regulatory T cells. In addition, NMP liver tissue was less necrotic and apoptotic compared to SCS-preserved livers, with less neutrophils infiltrating the periportal area (84).

Overall, with the clinical and experimental evidence gained so far for the field of SOT, dynamic preservation methods - and NMP in particular - provide the means to introduce organ conditioning so as to lower immunogenicity as well as pro-inflammatory markers while ensuring the promotion of graft regeneration.

## Immunomodulation During MP

Machine perfusion (MP) offers a previously non-existent link between basic and clinical science. The opportunity to treat an organ during preservation creates a new field of translational research. In this context, one of the main targets that can now be addressed is the modulation of the immunogenicity of a specific graft. In theory, such immunomodulation could allow the obviation of lifelong immunosuppression or even forge a path to the holy grail in transplantation—the achievement of tolerance. Importantly, MP offers a platform to apply immunomodulatory strategies that have been elucidated in *in vitro* or preclinical models in the past.

In the existing literature, three major such strategies have been investigated, which are discussed herein. One of the most promising is the application of stem or progenitor cells, with the potential to suppress immunogenicity and help repair injured tissue. Another strategy is the administration of anti-inflammatory/immunomodulatory drugs and agents, and, finally, gene transduction by adenoviral vector gene delivery.

The majority of studies have been carried out under normothermic conditions. However, oxygenated perfusion of a graft *per se* seems not only to protect against preservation injury but also to downregulate the immune system and blunt the alloimmune response, as was shown in a rodent model of hypothermic oxygenated perfusion (HOPE) (154). In particular, the constant flow of fluids in the vessels during MP is regarded to promote the expression of vasoprotective endothelial genes, alleviating the microcirculatory failure associated with ischemia-reperfusion injury (IRI) (155–157). On the other hand, when it comes to additional modification, it is perfusion under normothermic conditions that seems to represent the ideal platform, since NMP grants a physiological metabolic state (81, 158–160).

Many of the ongoing studies addressing the field of immunomodulation during MP are carried out in a porcine setting, as pigs have appropriate size and anatomy as well as immunologic characteristics (161). Whereas, much of our knowledge on organ preservation is derived from various animal studies (162), the porcine model above all has now evolved as an ideal model, making *ex vivo* porcine organ perfusion models a suitable platform for translational transplant research. According to a recent review, in 2017, 22 articles discussed *ex vivo* porcine organ perfusion within the context of transplant preservation surgery (162), but the number of articles has steadily increased since then. However, also in this setting, it is important to highlight potential limitations when translating experiences between species (158).

### Stem and Progenitor Cells

Several candidate cells have been investigated, including stromal mesenchymal cells (MSCs), induced adult pluripotent stem cells, fetal stem cells from placenta, membranes, amniotic fluid, and umbilical cord, and hematopoietic cells (163). Among these, MSCs have been reported to represent the most promising cell subset. MSCs are multipotent cells that are found in adult tissues, including adipose tissue and bone marrow, where they support function and repair. Importantly, they have been shown to abate immune and inflammatory responses via the release of paracrine effectors (164, 165).

This circumstance has led to a broad application of MSCs in a variety of pathologies. Therefore, a prerequisite for research on MSCs is the agreement on criteria that define these cells and allow comparability between studies. The International Society for Cellular Therapy has established the minimum criteria that a cell must meet to be considered an MSC: first, an MSC must be plastic-adherent when maintained in standard culture conditions; second, an MSC must express CD105, CD73, and CD90 and lack expression of CD45, CD34, CD14 or CD11b, CD79alpha or CD19, and HLA-DR surface molecules; third, an MSC must differentiate into cell types of mesodermal origin *in vitro* (166, 167).

The fact that MSCs are known for their potent anti-inflammatory and regenerative capacities, combined with the finding that a therapeutic effect can be achieved with either autologous or allogeneic MSCs (168), has led to the conduction of several studies investigating whether their application is feasible in the context of MP. However, the detailed mechanisms by which MSCs exert their anti-inflammatory and regenerative potential have not yet been depicted. The main mechanism of action is supposed to rely on secreted mediators. Therefore, an appropriate timeframe is likely to be required for these cells in order to mediate beneficial effects (169). This hypothesis is underlined by the findings of a recent study in a porcine *ex vivo* lung perfusion (EVLP) model. Twelve-hour NMP of DCD lungs with intravascular delivery of MSCs (150 × 10^6^) resulted in reduced levels of interleukin-8 (IL-8), a pro-inflammatory cytokine associated with reperfusion injury, along with increased levels of vascular endothelial growth factor (VEGF) (170). The fact that IL-8 was suppressed is of particular note, since MSCs are known to produce the anti-inflammatory cytokine IL-10, which in turn has been reported to significantly suppress the production of IL-8 in a dose-dependent manner (169).

In human kidneys, Brasile et al. demonstrated actual renal regeneration mediated by MSCs under 24 h of *ex vivo* normothermic perfusion. The authors used an exsanguinous metabolic support (EMS) tissue-engineering platform to study five pairs of kidney allografts from DCD donors. Whereas, one kidney of each pair solely underwent perfusion, the partner kidney was EMS perfused with MSC (1 × 10). Indeed, a reduced inflammatory response was observed along with increased synthesis of adenosine triphosphate and growth factors and a normalization of the cytoskeleton and mitosis (171).

Even though these findings are promising, more insight needs to be gained regarding the effects of the NMP milieu on MSC themselves and the comparability between human and porcine data. These aspects were highlighted in a kidney NMP model, showing that while the suspension conditions reduced the viability of porcine MSC by 40% in both perfusion fluid and culture medium, the viability of human MSC was reduced by suspension conditions by 15% in perfusion fluid, and no differences were found in survival in culture medium. Furthermore, it was shown that a freeze-thawing process impaired survival, metabolism, and the ability to adhere to endothelial cells. The authors concluded that NMP conditions affect MSC but show sufficient support of their function and survival that MSC administration through NMP should be considered, and secondly that slight differences in the behavior of porcine and human MSC need to be taken into account (158).

Another recent analysis focused on the characteristics of culture-expanded MSC, investigating heir viability and homin during NMP. Kidneys were perfused for 7 h in the presence of 10^5^, 10^6^, or 10^7^ human adipose tissue-derived MSC. Intact MSCs were detected in the lumen of glomerular capillaries, but only in the 10^7^ MSC group. After a rapid decline of cell numbers during NMP, only a small portion of the MSCs were intact, and these were clustered in a minority of glomeruli. Apart from outlining the complexity of MSC therapy during MP, the authors concluded that “*an exciting new window of opportunity might emerge to actively pre-condition isolated organs in a fully controlled setting and in the absence of an immune response, before they are transplanted”* (172).

It is noteworthy that such promising findings have recently led to the creation of the international “MePEP consortium” (173) in order to study this novel modality of treatment in preparation for human trials. Therefore, more findings on MSC and immunomodulation can be expected in the near future.

### Anti-inflammatory Agents

Alternatively, pharmacologic interventions to decrease immunogenicity or prevent recurrent disease can be applied during MP. The seemingly most obvious strategy to influence the inflammatory profile is the delivery of anti-inflammatory agents directly into the machine perfusion circuit, treating the liver *ex vivo* during the preservation period to obviate the need for lifelong immunosuppression or to improve long-term outcomes separate from the physiological quality of the organ at the time of transplantation (174).

Several targets for potential agents have been identified. Amongst these is TNF-alpha, one of the most potent proinflammatory cytokines, which is released in response to and has been implicated in the pathogenesis of IRI (175, 176). Therefore, in a recent clinical HMP study, it was hypothesized that the administration of the TNF-α inhibitor etanercept could improve outcomes following kidney transplantation. However, no significant differences were found concerning kidney machine perfusion parameters, including average flow and vascular resistance, nor did the authors observe significant changes regarding DGF, rejection episodes, or allograft survival (177). However, it has to be taken in mind that this study was carried out under hypothermic conditions. Although possible in other machine-perfusion techniques, NMP seems to be ideal, as active metabolism permits graft intervention and modification during preservation, circumstances that are not present in HMP (178).

More promising data come from a porcine study in which a variety of agents were added to act at different levels. Prostaglandin E1, a prostacyclin analog with vasodilator, antiplatelet, fibrinolytic, and several other anti-inflammatory properties, was continuously administered. In addition, n-acetylcysteine was added due to its free radical scavenging properties. Sevoflurane was administered due to its protective properties on endothelial cells, and carbon monoxide was added with the objective of improving vasodilatation and reducing inflammation. Indeed, during a 3-day follow-up after transplantation, this treatment resulted in lower AST levels as well as lower levels of IL-6, tumor necrosis factor α (TNF- α), and galactosidase and increased IL-10 levels (179).

Other candidate markers for modulation during MP are anti-inflammatory receptors. In this context, the adenosine A2A receptor downregulates inflammation, including the suppression of CD4^+^ and CD8^+^ T cells, and increases endothelial cell nitric oxide (174). Since experiences in rabbit experiments showed that adenosine A2A receptor activation can diminish IRI (180), a subsequent study evaluated whether treatment with an adenosine A2A receptor agonist could be beneficial during normothermic *ex vivo* lung perfusion (EVLP). Indeed, EVLP with targeted A2AR agonist treatment could attenuate IRI after transplantation of DCD donor lungs subjected to prolonged 12-h cold preservation in a preclinical porcine model (181).

### Adenoviral Vector Gene Delivery

As cellular metabolism is preserved during normothermic perfusion, it represents a potential platform for effective gene transduction in a specific graft (182). To test this hypothesis in donor lungs, Yeung et al. used a porcine model of EVLP and treated them with an E1-, E3-deleted adenoviral vector encoding either green fluorescent protein (GFP) or the immunosuppressive interleukin-10 (IL-10). They observed a decreased expression of inflammatory cytokines such as IL-1, TNF-α, and IL-6, as well as attenuation of the alloimmune response following transplantation (174, 182).

This finding is also of particular interest, since the delivery of adenoviral vectors could result in a prompt innate immune response by macrophages, recruiting circulating neutrophils, which in turn propagate the inflammatory response. Moreover, in the transplant setting, this preexisting inflammation could potentiate subsequent IRI (182, 183). Therefore, the authors compared *in vivo* and *ex vivo* administration and showed that donor lung is superior to *in vivo* delivery since it leads to less vector-associated inflammation (182).

Concerning the kidney, already in 2002, proof of principle of a similar technique had been reported by Brasile et al., using a recombinant adenovirus, Ad5, CMV5 GFP encoded with green fluorescence protein. They achieved effective transfection and synthesis during 24 h of *ex vivo* normothermic perfusion (184).

Only recently has a normothermic *ex vivo* organ perfusion delivery method for cardiac transplantation gene therapy been reported. Adenoviral vector transduction was utilized to deliver particles of an Adenoviral firefly luciferase vector with a cytomegalovirus (CMV) promotor to porcine donor hearts during NMP and prior to heterotopic implantation. Along with a high copy number of vector genomic DNA in transplanted hearts, there was no evidence of vector DNA in either the recipient's native heart or liver, substantiating the applicability and safety of the protocol.

These findings make it likely that this technology could be feasible for other organ systems as well. A wide variety of interesting genes could be targeted. As has been reviewed lately (159), promising possibilities arise when translating the findings of rodent models using adenoviruses expressing CTLA4Ig (185) to the setting of NMP; also, NMP could be used to deliver gene therapies that induce cytoprotection against IRI, such as myr-Akt (159, 186).

Outstandingly, Figueiredo et al. recently showed that antigen silencing is feasible during NMP. In a porcine model of lung NMP, short hairpin RNAs were delivered by lentiviral vectors, successfully reducing the immunogenicity of the lung by silencing MHC expression on the endothelium. The authors concluded that the decrease in immunogenicity carries the potential to generate immunologically invisible organs to counteract the burden of rejection and immunosuppression (187).

### Organ Reconditioning and Repair

Recent experimental data primarily focusing on marginal livers furthermore suggested that NMP offers a platform for organ reconditioning and repair.

Impressive data from Birmingham, UK, showed that livers discarded due to pronounced steatosis could be effectively treated with defatting agents during 6 h of NMP. Tissue triglycerides were lowered by 38% and macrovesicular steatosis by 40%, which was associated with a down-regulation of inflammatory marker expression relevant for oxidative injury and activation of immune cells (CD14; CD11b), combined with reduced inflammatory cytokines in the perfusate (TNFα, IL-1β) (188). In addition, arterial vasospasm can be modulated by the application of ET-1 antagonists, prostacyclin analog, or calcium channel antagonists during NMP (189). Furthermore, NMP can be used for the direct and efficient application of antiviral agents, as shown in a porcine NMP model, in which Miravirsen, an inhibitor of hepatitis C virus (HCV) replication, produced improved uptake compared to SCS control livers. The authors concluded that this approach might offer a future strategy to prevent HCV reinfection after liver transplantation (190). Likewise, HCV-infected human donor lungs could effectively be treated during short-time EVLP via physical viral clearance combined with germicidal light-based therapies (191).

In summary, immunomodulation in the context of MP seems to offer a plethora of novel strategies. A series of porcine and human studies distinctively underline its effectiveness and feasibility. However, as formulated in a recent review (191, 192), the studies conducted so far just scratch the surface of conceivable interventions. In particular, NMP provides an ideal platform for immunomodulatory modifications. The next step will be the translation of findings from preclinical and rodent models as well as HMP trials into the setting of NMP.

## The Immediate Future in the Transplantation of Marginal Organs

Much speculation about the changes in transplantation is currently energizing the field. The conceptual approach of extracorporal organ preservation and monitoring begs for speculation about organ reconditioning, repair, and treatment. While these are all valid considerations and perfectly reasonable hopes, the implementation of machine perfusion is the first step. Immediate and widespread adoption of HMP and NMP is unlikely since this technology does not immediately improve the outcome with regard to the primary endpoints in transplantation: patient and graft survival. Authority approval and market entry are largely based on secondary endpoints such as delayed graft function (DGF, kidney) or primary poor function (PPF, liver), which are considered to serve as surrogates for improved long-term results. The conceptual limitation with surrogates is, however, that the predictive value is not uniformly robust or formally established and immediately reproducible. The DGF rate, for example, in kidneys from DCD donors is high, but the predictive value of DFG in these cases is low (193). The definition of PPF is mostly based on high AST/ALT values early after transplantation. While these parameters indicate hepatocyte damage, correlation with the eventual outcome is very limited (194). Hence, the adoption of the technology is possibly built on the wish for innovation and the belief in the value of the time gain with NMP. Preliminary data on HOPE in recent and ongoing trials indicate a possible benefit in organ survival. If such an outcome is eventually formally achieved, the arguments for implementation will be more substantial.

For clinical implementation, several regional and center factors play an important role. First, the retrieval team may be different from the team eventually performing the transplantation. Even if the retrieval team is familiar with the technology, the preparation of the graft prior to NMP (more than HMP) is more substantial and more definitive for the fate of the graft. Hence, in a system with longer-distance organ exchange, additional steps and a new routine would need to be established. The technology and the shifting of the work of the backtable procedure to the retrieving team need to be considered. The alternative approach where the organ is transferred to the recipient center and is then machine perfused is currently being pursued, but data indicating the actual advantage of this approach are lacking (195). For now, NMP serves as a technology that allows one important asset in surgery to be gained: time. The value of time and flexibility is dependent on the regional circumstances but may hold great potential for easing the surgery logistics, working hours, and surgical training but also gives rise to higher risks of nighttime procedures. The actual value of time in this context, however, requires further attention and needs to be better defined.

The consideration of longer-term organ preservation under normothermic conditions further carries the challenge of defining the responsibilities for managing and monitoring the organs. Eventually, the working place description of health care workers involved in this will require adoption and formal training. Standard operating procedures (SOP) and safety parameters need to be established for a wider spread use of the technology. It is likely that the advanced technological requirements and the need for 24-h availability of knowhow and personnel would favor the establishment of regional hubs for MP.

Despite the hype in the field, the clinical adoption of MP is slow. In addition to the above-mentioned factors, the lack of reimbursement is a stringent limitation in many regions. For the technology to be reimbursed, the authorities will require hard facts. Hence, the path to more widespread clinical use might be long, and the focus should remain on the demonstration of superiority regarding the most relevant clinical endpoints.

One major important step toward collective advancement in this field would be the definition of data points during and after MP and the establishment of registries for coordinated data collection. Such data points should include the definition and terminology of the various time points and actions such as cold flush and storage prior to MP, temperature and flow during MP, parameters indicating metabolic function and bile/urine production during MP, second flush, second cold ischemia time, and others. One of the hurdles in improving NMP is the large number of variables added to preservation. Identifying the relevance of individual parameters will require attention to the details of the procedure and adequate data collection and handling. To the knowledge of the authors, no routine data collection and no consensus toward data points have been established at present.

The decision-making process in MP is relatively arbitrary since the data collected during MP are suggestive but not formally established as quality-defining parameters. Since the decision to transplant an organ or not is extremely meaningful, great care needs to be applied in the process, and detailed documentation of the reasoning for decision making should be carried out. A greater effort toward orchestrated data collection could help to streamline and eventually enhance the robustness of the decision-making process.

The preference for MP to be used for the preservation of marginal organs results from the greater need for organ assessment and the greater potential benefit of better preservation. This might be particularly true and relevant for organs from uncontrolled DCDs, where the circumstances and the accumulated damage to the organs might be less clear. The value of an additional assessment under these conditions is not only meaningful with respect to the number of additional organs for transplantation but also for preventing the transplantation of organs that are severely damaged. While the assessment of this subject would be highly valuable and is much needed, the behavior of the investigators in the NMP trial by Nasralla et al. indicates the limitations and conflicts observed with this approach (62). Since the trial cannot be fully blinded, the bias generated by the fact that a technology is used that is deemed superior and the data generated during MP define a deviation in the behavior of the decision makers.

## The Far Future of (Marginal) Organ Transplantation

The fantasies building on the realization of extracorporal organ preservation under physiological conditions are currently fuelling hopes that this technology could facilitate tissue regeneration, organ repair, immunomodulation, xenograft humanification, and many other things. MP as a platform may impact medicine far beyond transplantation and delivers a unique chance to alter the treatment of organ failure and organ disease. Between the imagination and the realization of medical advancement stands a mountain of work and an as yet unknown but probably large number of technical and methodological challenges. An important initial goal is the expansion of the duration of MP and the establishment of an equilibrium of the condition of the organ. Preservation of organs for several days will likely require additional modifications from the currently existing technologies. Preservation of the acid-base equilibrium, nutrition organ weight-induced pressure, electrolyte shift, hemolysis, and many other challenges may require closer attention. A tissue- and/or cell-specific and targeted treatment is a realistic consideration, and proof of concept trials indicate the feasibility of this approach (196, 197). The treatment and replacement of damaged or displaced elements of organs may evolve as a new discipline and help the field to make the leap to serve one of the greatest unmet needs: The effective treatment of damaged organs.

## Author Contributions

TR and SS conceived and designed the study and wrote the manuscript. BC, RO, AW, JD, CK, and CB wrote sections of the manuscript. DO and MG helped to revise the manuscript, tables and figures. All the authors contributed to manuscript revision, read, and approved the submitted version.

## Conflict of Interest

The authors declare that the research was conducted in the absence of any commercial or financial relationships that could be construed as a potential conflict of interest.

## Abbreviations

A2AR: Adenosine A2A receptor
AST: Aspartate aminotransferase
ATP: Adenosine triphosphate
CIT: Cold ischemia time
CMV: Cytomegalovirus
CVA: Cerebrovascular accident
DAMP: Danger-associated molecular pattern
DBD: Donation after brain death
DC: Dendritic cell
DCD: Donation after cardiac death
DGF: Delayed graft function
D-HOPE: Dual hypothermic oxygenated perfusion
DRI: Donor Risk Index
EAD: Early allograft dysfunction
ECD: Expanded criteria donor
ECMO: Extracorporal membrane oxygenation
EMS: Exsanguinous metabolic support
ESP: Eurotransplant senior program
ET-1: Endothelin-1
EVLP: *Ex vivo* lung perfusion
FABP: Fatty Acid- Binding Protein 1
GFP: Green fluorescent protein
GST: Glutathione S-Tranferases
H-FABP: Heart-type fatty acid-binding protein
HOPE: Hypothermic oxygenated perfusion
HMP: Hypothermic machine perfusion
HMPO: Oxygenated hypothermic machine perfusion
IFNγ: Interferon gamma
IL-10: Interleukin-10
IRI: Ischemia-reperfusion injury
KDRI: Kidney Donor Risk Index
LDH: Lactate dehydrogenase
MP: Machine perfusion
MSC: Mesenchymal stromal cells
NAG: N-acetyl-beta-D-glucosaminidase
NMP: Normothermic machine perfusion
NK: Natural killer cell
NKT: Natural killer T cell
NRP: Normothermic regional perfusion
OCS: Transmedic Organ Care System
PDR: Pancreas Donor Risk Index
PGD3: Prostaglandin D3
PNF: Primary non-function
PV: Pressure volume
RCT: Randomized controlled trial
ROS: Reactive oxygen species
SCD: Standard criteria donor
SCS: Static cold storage
SOFT: Survival Outcomes Following Liver Transplantation score
SOP: Standard operating procedure
SOT: Solid organ transplantation
TNF-α: Tumor necrosis factor α
UNOS: United Network for Organ Sharing
VEGF: Vascular endothelial growth factor
WIT: Warm ischemia time.
